# Kinetic Models of Secondary Active Transporters

**DOI:** 10.3390/ijms20215365

**Published:** 2019-10-28

**Authors:** Verena Burtscher, Klaus Schicker, Michael Freissmuth, Walter Sandtner

**Affiliations:** Institute of Pharmacology and the Gaston H. Glock Research Laboratories for Exploratory Drug Development, Center of Physiology and Pharmacology, Medical University of Vienna, 1090 Vienna, Austria; verena.burtscher@meduniwien.ac.at (V.B.); klaus.schicker@meduniwien.ac.at (K.S.); walter.sandtner@meduniwien.ac.at (W.S.)

**Keywords:** secondary active transporters, serotonin transporter, kinetic models, solute carrier, energy coupling, steady-state substrate uptake, concentrative power

## Abstract

Kinetic models have been employed to understand the logic of substrate transport through transporters of the Solute Carrier (SLC) family. All SLC transporters operate according to the alternate access model, which posits that substrate transport occurs in a closed loop of partial reactions (i.e., a transport cycle). Kinetic models can help to find realistic estimates for conformational transitions between individual states of the transport cycle. When constrained by experimental results, kinetic models can faithfully describe the function of a candidate transporter at a pre-steady state. In addition, we show that kinetic models can accurately predict the intra- and extracellular substrate concentrations maintained by the transporter at a steady state, even under the premise of loose coupling between the electrochemical gradient of the driving ion and of the substrate. We define the criteria for the design of a credible kinetic model of the SLC transporter. Parsimony is the guiding principle of kinetic modeling. We argue, however, that the level of acceptable parsimony is limited by the need to account for the substrate gradient established by a secondary active transporter, and for random order binding of co-substrates and substrate. Random order binding has consistently been observed in transporters of the SLC group.

## 1. Introduction

Biological membranes are diffusion barriers for polar solutes. The solute carrier (SLC) group of membrane proteins allows for the passage of polar solutes across the cell surface membranes and through intracellular membranes [1]. By virtue of this action, SLCs play a central role in maintaining cell homeostasis, in supporting metabolism and neurotransmission and in extruding toxic compounds [2,3,4]. The solute carrier proteins include examples of transporters, which by lowering the membrane-imposed diffusion barrier, facilitate the downhill flow of solutes along their electrochemical gradient, i.e., facilitating transporters. Facilitating transporters are referred to as equilibrative, (as opposed to concentrative), transporters, to indicate that (unless the substrate carries a charge), the concentration of substrate on both sides of the membrane will be the same at the steady state. The other type of membrane transporters in the SLC group are secondary active transporters. These also lower the diffusion barrier for polar solutes, but in addition allow solutes to flow uphill against their electrochemical gradient by coupling their transport to the gradient of a second solute [5]. In most instances, this second solute is an ion such as Na^+^ or H^+^ [6,7], but there are also examples of transporters which use the gradient of small, organic molecules, such as lactate, to drive concentrative solute/substrate transport [8]. Secondary active transporters can operate in two ways: In symporters, the vectorial transport of substrate and co-substrate is in the same direction; in antiporters, the substrate and co-substrate are counter-transported.

It is well established that SLC transporters operate according to the alternate access mechanism [9]: The transporter presents a substrate-binding site to the extracellular side of the membrane; upon binding of substrate and co-substrates (e.g., Na^+^ and Cl^−^), conformational changes occlude the extracellular access pathway and expose the solutes to the intracellular space. This results in substrate translocation into the cell, which is followed by the return of the empty transporter (or of the transporter liganded to a counter-transported solute) to the outward-facing conformation. At this point, a new substrate molecule can bind, and the same process can repeat all over. This sequence of events constitutes the transport cycle, which can be viewed as a closed loop of partial reactions (i.e., binding reactions and conformational changes). It is gratifying to note that crystallographic investigations of bacterial and mammalian transporters, in conjunction with computational modeling, have provided compelling evidence in support of this general concept, regardless of whether the transporter operates via a rocking bundle [10] or an elevator mechanism [11,12,13].

In the present study, we highlight the usefulness of kinetic models in the description of the transport cycle of secondary active transporters. Kinetic models can help to find realistic estimates for conformational transitions between individual states. This allows for quantitative predictions of the amount of substrate flux through transporters under initial conditions and at a steady state. Importantly, kinetic models provide insights into how secondary active transporters utilize the electrochemical gradient to establish and maintain a substrate gradient. Moreover, we show that they also allow for predicting the intra- and extracellular substrate concentrations maintained by a transporter at steady state, when conversion efficiency between the electrochemical gradient of the driving solute and the substrate is low.

Finally, we define the criteria for the design of a credible kinetic model of a secondary active transporter. Parsimony is the guiding principle of kinetic modeling to avoid overparameterization. However, experimental observations often impose limits to the level of acceptable parsimony. Here, we show how an inclusion or omission of specific transporter features impinge on the ability of a kinetic model to faithfully account for experimental outcomes. We focus on transporters of the SLC6 family. However, our results also apply to other SLC families.

## 2. Results and Discussion

### 2.1. Kinetic Models can Faithfully Predict Intra- and Extracellular Substrate Concentrations Maintained by Secondary Active Transporter at Steady State

**Figure 1:**
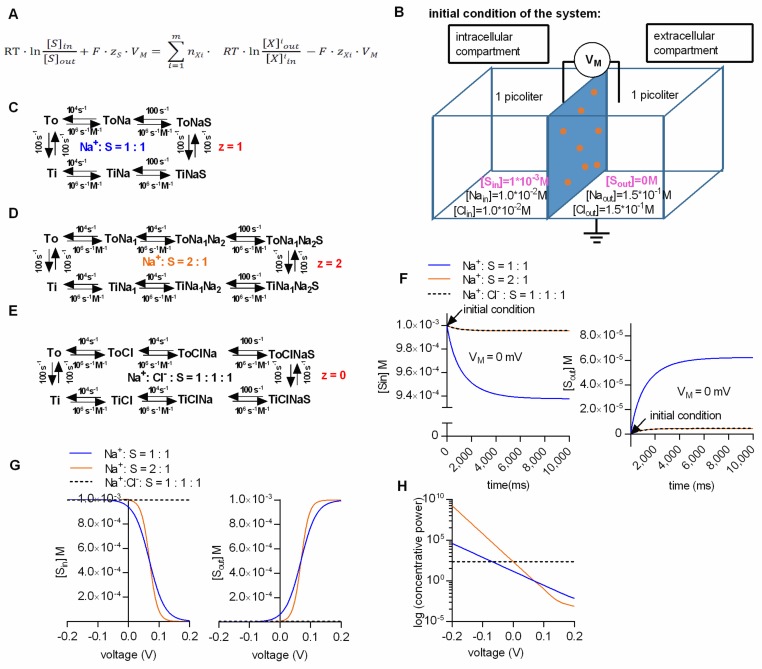
Kinetic models can correctly predict the substrate concentrations maintained by a secondary active transporter at steady state. (**A**) Generalized equation of the electrochemical potential of a secondary active transporter. T is the temperature in Kelvin, R the gas constant and F the Faraday constant. The symbols z_S_ and z_Xi_ are the valences of the substrate and the i’th ion species, respectively. Next, m is the number of ion species used by a transporter to support concentrative substrate uptake, and n_Xi_ is the number of dissipated ions per cycle belonging to one ion species. (**B**) The two compartment-model, (i.e., intra- and extracellular- with volumes of 1 pl each) separated by an impermeable membrane containing 10^6^ transporters. Indicated are the intra- and extracellular ion and substrate concentrations at the start of the simulation (i.e., initial condition). V_M_ is the voltage difference between the intra- and extracellular bulk solution. (**C**–**E**) Kinetic models for different transport stoichiometries: Na:S = 1:1 (in blue), Na:S = 2:1 (in orange), Na:Cl:S = 1:1:1 (in black). (**F**) Intra- and extracellular substrate concentrations (left and right panel, respectively) as a function of time for three transporter stoichiometries. The membrane potential in the shown simulation was set to 0 mV. The arrows mark initial conditions. (**G**) Intra-and extracellular substrate concentrations (left and right panel, respectively) as a function of voltage for the three transporter stoichiometries. (**H**) Concentrative power of the three stoichiometries as a function of voltage. The concentrative power (S_in_/S_out_) was plotted on a logarithmic scale (*y*-axis) to allow for a better comparison between stoichiometries.

Secondary active transporters are molecular machines, which like any other machine, convert one form of energy into another: SLC6 transporters, for instance, use the electrochemical potential of an ion (e.g., Na^+^) to establish and maintain a substrate gradient [5]. The generalized equation for the electrochemical potential of secondary active transporters (Figure 1A) allows for calculating the intra- and extracellular substrate concentrations maintained by a transporter at the steady state, given that the transport stoichiometry, the intra- and extracellular ion concentrations, and the membrane potential, are known. What is striking, however, are the factors which are not included in the equation, and which hence have no influence upon the substrate concentrations at the steady state. For instance, in the equation there is no reference to ion or substrate affinities for the transporter. Moreover, the equation is indifferent to the direction of transporter insertion into the membrane. In fact, the equation implies that inverting the transporter (i.e., inserting the transporter into the membrane with its extracellular face in the cytosol) would not affect the resulting steady-state concentrations of the substrate. In addition—but less surprising—the thermodynamic equation does not take the kinetics (e.g., the transport rate) of the transporter into account.

While substrate uptake by the transporters at steady state is quantitatively described by the equation of the electrochemical potential, kinetic models are required to faithfully describe the transport rates at the pre-steady state [14,15]. Accordingly, substrate uptake at steady state and transport kinetics have typically been treated as separate matters. Here we aimed at closing this gap by assessing the ability of kinetic models to also predict intra- and extracellular substrate concentrations at this steady state.

We calculated the steady-state concentrations with the kinetic model by assuming two compartments (one intra- and one extracellular) which were separated by an impermeable membrane containing 10^6^ transporters (Figure 1B). The volume of the intra- and extracellular compartment was chosen to be 1 pl each. For the simulation we further assumed (i) that all of the substrate was initially contained in the intracellular compartment (we set the intracellular substrate concentration to 1 mM), and (ii) that the substrate did not carry a charge. We tested this model by running simulations for three different transport stoichiometries (i.e., Na^+^/S, 2Na^+^/S and Na^+^/Cl^−^/S in the panels C, D and E, respectively, of Figure 1), and by comparing the resulting concentrations with the calculated values from the thermodynamic equation in Figure 1A. We stress that in these calculations (according to the reaction schemes outlined in panels C, D and E of Figure 1), we assume a hypothetical transporter: In Figure 1 and all subsequent figures, we chose to use rate constants, which describe the GlyT1 [16], but it is obvious that the stoichiometries were varied to an extent, which is not recapitulated by the experimental data for GlyT1.

Figure 1F shows the substrate concentration in the intra- and extracellular compartment as a function of time in the left and right panel, respectively. In the simulation we assumed the presence of physiological ion concentrations (i.e., 150 mM NaCl extracellularly and 10 mM NaCl intracellularly) and a membrane potential of 0 mV. At this voltage, substrate transport is solely driven by the chemical potential (i.e., the ion gradients). Plotted are the results for the three alternative stoichiometries. The intracellular substrate concentrations dropped initially, because all substrate was contained in the intracellular compartment. Concomitantly, the extracellular concentration rose as the substrate entered the extracellular compartment via the transporter. As predicted for the 0 mV transmembrane potential, the lowest concentrative power (i.e., the ratio of intracellular to extracellular substrate concentration) was seen with the stoichiometry Na^+^/S (blue trace). More importantly, regardless of the stoichiometry, both the intra- and the extracellular substrate concentration reached a steady-state level after a few seconds. The substrate concentrations at this equilibrium were in excellent agreement with the concentrations calculated from the thermodynamic equation. We note that it is not always possible to correctly determine the equilibrium concentrations: Initial substrate accumulation may overshoot, such that the apparent steady-state equilibrium is lower than the initial peak uptake. This is accounted for by the rapid dissipation of the ionic gradient. When corrected for the change in driving force, estimates for the equilibrium concentrations can nevertheless be extracted from the data [17].

The Na^+^/S and the 2Na^+^/S stoichiometry are both electrogenic, while the Na^+^/Cl^−^/S stoichiometry is electroneutral. Figure 1G shows the simulated intra- and extracellular substrate concentration at steady state as a function of voltage in the left and right panel, respectively. In the Figure 1H we plotted the concentrative power (S_in_/S_out_) as a function of voltage for the three stoichiometries. As expected, when assuming an electroneutral stoichiometry (i.e., Na^+^/Cl^−^/S), the intra- and extracellular substrate concentrations (black dashed line) did not change with voltage. In contrast, in the case of the two other stoichiometries, the intra- and extracellular substrate concentrations displayed a pronounced voltage dependence. When we compared the results of the simulation with the values calculated from the thermodynamic equation, we also found them in excellent agreement. These results confirmed that our model can faithfully predict the intra- and extracellular substrate concentration maintained by a secondary active transporter at steady state.

### 2.2. Loose Coupling between Ions and the Substrate

The efficiency of energy conversion is described by the ratio between the useful output of an energy conversion machine and the input (i.e., the energy conversion efficiency η). The above kinetic models and the thermodynamic equation implicitly assume that η is 1, which is the maximal value that η can adopt. Larger values, as would be required to create a perpetual motion machine, are thermodynamically forbidden. Yet, for many energy conversion machines (e.g., Carnot machines [18]) the theoretical limit for η is much lower than 1.

However, to the best of our knowledge, there is no theoretical limit for η in the case of secondary active transporters. Hence, η can conceivably be 1, or can at least adopt values very close to 1.

**Figure 2:**
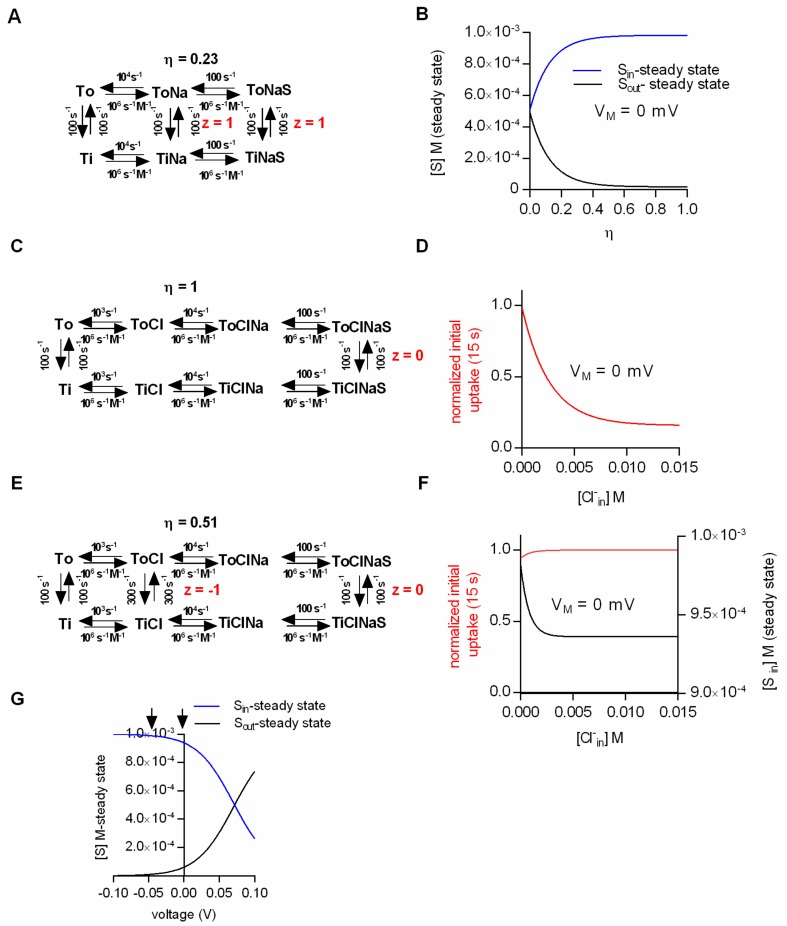
Substrate transport at low conversion efficiencies. (**A**) Scheme of a transport model with low Na^+^/substrate conversion efficiency (η = 0.23). The kinetic model allows for Na^+^ slippage. (**B**) Steady-state substrate concentrations as a function of the conversion efficiency (η). The membrane potential in the simulation was set to 0 mV. (**C**) Scheme of a transport model in which the conversion efficiency (η) is 1. (**D**) Simulated normalized initial uptake as a function of the intracellular Cl^−^ concentration for the scheme in **C.** V_M_ in the simulation was 0 mV. (**E**) Scheme of a model for which the coupling efficiency (η) is 0.51. The model allows for Cl^−^ slippage, but fully utilizes the electrochemical potential of Na^+^. (**F**) Simulated normalized initial (red line) and steady-state uptake (black line) as a function of the intracellular Cl^−^ concentration at 0 mV. (**G**) Intra- and extracellular substrate concentrations at the steady state as a function of voltage calculated for the scheme in E. The arrows indicate the voltages that were compared in the vesicular preparation [20].

The ability of the kinetic model to faithfully predict intra- and extracellular substrate concentrations at steady state prompted us to ask the following question: Can kinetic models correctly predict the substrate concentrations maintained by the secondary active transporter at a steady state under the assumption that the electrochemical potential of the ion and the substrate are only partially coupled (η < 1)? We addressed this question by positing the loose coupling of Na^+^ and substrate (Figure 2A). In contrast to the original scheme (Figure 1C), the scheme in Figure 2A allows for the conversion of the transporter from the outward- to the inward-facing conformation in the absence of the substrate. This gives rise to Na^+^ slippage. The calculated Na^+^/substrate conversion efficiency in the example shown was η = 0.23 (the procedure to determine η is described in the methods section, *cf*. equation 8). In Figure 2B we plotted the steady-state substrate concentrations as a function of the conversion efficiency. The membrane potential in this simulation was set to 0 mV. At a conversion efficiency of 1, the simulated intra- and extracellular substrate concentrations matched the prediction of the thermodynamic equation (Figure 1A). However, at successively lower conversion efficiencies the values obtained from the simulation increasingly deviated, such that at η < 0.1 there was little concentrative substrate uptake. As expected, at η = 0, the substrate concentrations on both sides of the membrane were exactly equal. We want to emphasize, however, that the complete absence of concentrative substrate uptake at η = 0 was not caused by the dissipation of the Na^+^ gradient. In the simulation we kept the Na^+^ gradient constant at all times, as would also be the case in biological cells, in which the Na^+^/K^+^ pump is abundantly expressed. The sole reason why there was no concentrative uptake is that the transporter can no longer harvest the energy contained in the Na^+^ gradient to drive the concentrative substrate uptake. Hill [5] described an analytical approach to calculate the extra- and intracellular substrate concentrations at steady state also for models in which the substrate and the driving ions are only partially coupled ( see Materials and Methods).

When we compared the analytical solution with the results obtained from the simulations, we found them in excellent agreement. This provides confidence that kinetic models of secondary active transporters are useful to investigate the impact of loose coupling on the intra- and extracellular substrate concentrations at the steady state. The equation for the analytical solution can be readily derived in simple schemes like those shown in Figure 2A or Figure 2E, but this becomes substantially more challenging in complex models. However, these complex models, which encompass many additional loops, are required to explain experimental observations [16,19]. Thus, for more complex models, the simulations rather than the analytical solutions are the more practical approach to assess the effect of slippage on the intra- and extracellular substrate concentrations at the steady state.

### 2.3. Does SERT Utilize only a Fraction of the Energy Contained in the Transmembrane Cl^−^ Gradient for Concentrative 5-HT Uptake?

Electrophysiological data are available for a large collection of sodium-dependent SLC6 transporters [16,19,21,22,23,24]. Based on these data, Na^+^/substrate coupling in SLC6 transporters appears to be quite efficient. This is supported by the fact that Na^+^ slippage, which is expected to manifest as a leak current in the absence of the substrate, is rarely observed: There is, for instance, no indication for the presence of a substrate-independent leak current in the case of the transporters for GABA (GAT-1 to GAT-3) or for glycine (GlyT-1 and GlyT2). However, the monoamine transporters for serotonin (SERT), dopamine (DAT) and norepinephrine (NET) are known exceptions. These carry a current in the absence of the substrate, which can be blocked by selective inhibitors. In the case of SERT, it is believed that this leak current is carried by a channel mode, which SERT occasionally adopts, and which is thought to conduct Na^+^ [25]. In this context, it is worth noting that a channel mode, even if it dissipates the Na^+^ gradient, has only little influence on the coupling efficiency of a transporter. However, regardless of whether the leak current through SERT is produced by Na^+^ slippage or by the transporter entering a channel mode, its small amplitude (approx. 5% of the amplitude of the substrate induced current) suggests that the Na^+^/substrate conversion efficiency (η) in SERT is high (i.e., >0.95) [23]. Based upon published data, the same argument can be made for the two other monoamine transporters [26,27].

Many sodium-dependent SLC6 transporters require the presence of extracellular Cl^−^ for substrate transport. A non-comprehensive list of examples includes the transporters for taurine (TauT), creatine (CrT-1), GAT-1 to GAT-3, SERT, DAT and NET [19,28,29,30]. It is thought that these transporters have the ability to harvest the energy contained in the existing transmembrane Cl^−^ gradients to drive cellular substrate uptake. In a seminal study, Rudnick and coworkers showed that in membrane vesicles containing SERT, elimination of the Cl^−^ gradient reduced the uptake of 5-HT at the steady state by about 50% [19]. This finding is consistent with the idea that the Cl^−^ gradient is energetically coupled to the gradient of the substrate. However, the same study also showed that the initial substrate uptake (defined as 5-HT uptake in the first 15 s) was not affected by erasing the Cl^−^ gradient. In fact, later studies showed that initial substrate uptake is slightly increased when the intracellular Cl^−^ concentration is in the 100 mM range [21,31]. This inability of intracellular Cl^−^ to inhibit the initial substrate uptake is consistent with the observation that the presence of high intracellular Cl^−^ does not result in the reduction of the amplitude of the substrate-induced current carried by SERT [21].

We resorted to kinetic modeling to explore whether efficient Cl^−^/substrate coupling accounted for the absence of substrate uptake inhibition under initial conditions. Figure 2C shows the scheme of a model with a conversion efficiency (η) of 1. We employed this model to simulate the initial substrate uptake. In Figure 2D we plotted the normalized initial uptake as a function of the intracellular Cl^−^ concentration. As can be seen, the model predicts that raising the intracellular Cl^−^ concentration inhibits the initial substrate uptake. This, however, is inconsistent with the experimental evidence [20,21,31]. With η = 1, the transporter can only return to the outward-facing conformation in its empty form (Ti to To), but not with Cl^−^ bound. High intracellular Cl^−^ concentrations must result in a rebinding of Cl^−^ to the transporter, which ought to hamper progress through the transport cycle. This must reduce the initial substrate uptake and the amplitude of the substrate-induced current. Hence, we surmised that these seemingly contradicting observations—i.e., increase in initial but reduction in steady-state substrate uptake at high intracellular Cl^−^—can be explained by the partial coupling of the electrochemical potential of Cl^−^ to that of the substrate. To test this conjecture we employed the kinetic model shown in Figure 2E. In the displayed scheme the coupling efficiency (η) is 0.51. In Figure 2F we plotted the simulated initial and steady-state uptake as a function of the intracellular Cl^−^ concentration. It is obvious that the synthetic data recapitulated the reported findings in SERT (i.e., an increase of the initial substrate uptake upon raising intracellular Cl^−^ and a reduction of steady-state substrate uptake over the same range of intracellular Cl^−^ concentrations).

Nelson and Rudnick showed that substrate transport by SERT at the steady state was not affected by voltage [20]. The pertinent experiments were conducted on vesicles prepared from platelets. Valinomycin was employed to establish an intracellular-negative membrane potential (i.e., −50 mV): Vesicular 5-HT uptake was essentially the same in the presence and absence of the potential. We addressed the question whether these findings were consistent with Cl^−^ slippage: Figure 2G shows the intra- and extracellular substrate concentrations at steady state as a function of voltage (based on the model shown in Figure 2E, which allowed for Cl^−^ slippage). It is evident from Figure 2G that voltage has an impact on the steady-state uptake of the substrate. However, the predicted effect of voltage on substrate uptake was not detectable by the experimental approach of Rudnick and coworkers. The arrows in Figure 2G indicate the two voltages that were compared in the vesicular preparation. In order to see an effect of voltage on substrate uptake, it would have been necessary to assess a more positive voltage range. This, however, was not possible due to technical limitations, which are intrinsic to voltage control with potassium gradients and valinomycin. Based on these simulations, it is safe to conclude that in SERT the electrochemical potential of Cl^−^ and substrate are only partially coupled. Cl^−^ slippage is also the most plausible explanation to account for all experimental findings and to resolve the apparent discrepancies therein.

### 2.4. Partial Coupling between Ions and Substrate Renders the Intra- and Extracellular Substrate Concentration at Steady State dependent on the Kinetics of the Transporter.

**Figure 3:**
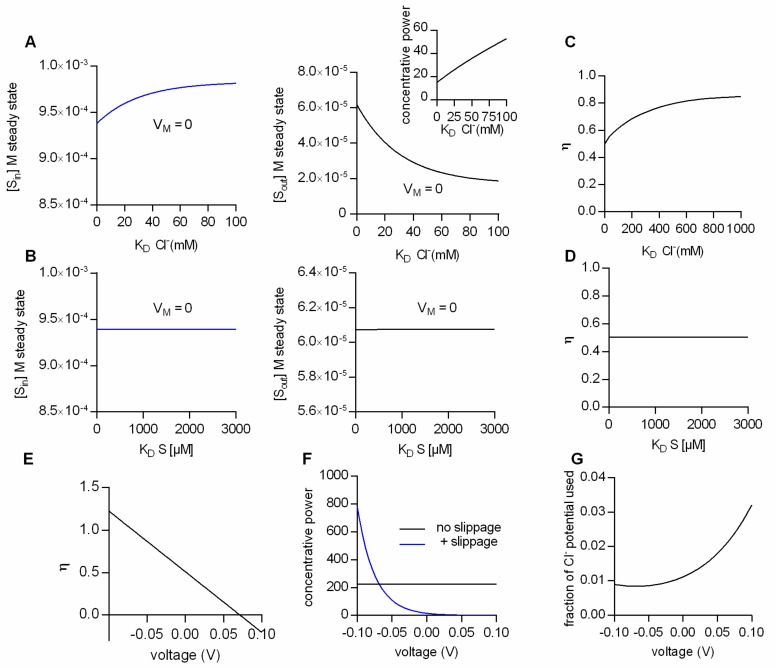
Impact of transporter kinetics on substrate concentrations at steady state at low conversion efficiencies. The simulated data in (**A**–**F**) are all obtained from the model displayed in Figure 2E. (**A**) Intra- and extracellular substrate concentrations at steady state as a function of the K_D_ for Cl^−^ (S_in_ is shown in the left and S_out_ in the right panel). V_M_ in the simulation was set to 0 mV. (**B**) Intra- and extracellular substrate concentrations at steady state as a function of the K_D_ for substrate, in the left and right panel, respectively. V_M_ in the simulation was 0 mV. (**C**) Conversion efficiencies (η) at 0 mV as a function of the K_D_ for Cl^−^. (**D**) Conversion efficiencies (η) at 0 mV as a function of the K_D_ for the substrate. (**E**) Ill-defined conversion efficiencies as a function of voltage. (**F**) Concentrative power as a function of voltage. Comparison between the fully-coupled model (black line-model scheme in Figure 2C) and the model with Cl^−^ slippage (blue line-model scheme in Figure 2E). (**G**) “fraction of the Cl^−^ potential used” (see definition in the Section 3 “Materials and Methods”) as a function of voltage.

As mentioned earlier, the intra- and extracellular substrate concentrations maintained by a fully-coupled transporter (i.e., with η = 1) at steady state are solely determined by the transport stoichiometry, intra- and extracellular ion concentrations and the membrane potential. This, however, cannot be true for a transporter operating with η values lower than 1. We employed the two-compartment model of Figure 1B, together with the kinetic scheme displayed in Figure 2E to identify the parameters, which influence the intra- and extracellular steady-state substrate concentrations at low conversion efficiencies. Figure 3A summarizes the results from simulations, where the ion concentrations were in the physiological range, the membrane potential was set to 0 mV and the Cl^−^ affinity of the transporter was varied: The right and left hand plot show the steady-state substrate concentration in the intra- and extracellular space, respectively, as a function of the K_D_ for Cl^−^. The model predicts that at lower Cl^−^ affinities (i.e., higher K_D_s) the concentrative power for substrate uptake is increased (see inset in Figure 3A). The synthetic data therefore imply that, with partial coupling, ion affinities can become a defining factor for the substrate concentrations maintained by a transporter at the steady state. In a second set of simulations we explored the impact of substrate affinity on the intra- (left hand graph in Figure 3B) and extracellular substrate concentrations at steady state (right hand graph Figure 3B): It is obvious from the data that the substrate affinity is immaterial to the substrate concentrations maintained at steady state.

Why is it that the steady-state distribution of substrate (concentrative power) is sensitive to the Cl^−^ affinity, but insensitive to the affinity of transport for the substrate? The answer to this question is provided in Figure 3C,D. In Figure 3C,D we plotted the calculated η values as a function of the K_D_ of Cl^−^ and of the K_D_ of the substrate, respectively. As can be seen, the η values change as a function of the K_D_ of Cl^−^, but they are not affected by a change in the K_D_ of the substrate. This can be rationalized as follows: The conversion efficiency is determined by the extent of ion slippage. At high Cl^−^ affinities, Cl^−^ is more likely to stay bound to the transporter. This favors the return of the transporter to the outward-facing conformation with the Cl^−^ bound (i.e., TiCl to ToCl). The latter gives rise to an increase in Cl^−^ slippage and concomitantly to lower η values.

The above consideration implies that the factors which determine η are model-specific. For instance, on inspection of the kinetic model in Figure 2E, it seemed likely that also voltage is a defining factor of η, because Cl^−^ slippage is assumed to be voltage-dependent, but the return of the empty transporter is not. Figure 3E shows η as a function of membrane voltage. It is evident that V_M_ is a determining factor of η. Oddly, however, the calculated conversion efficiencies have values more positive than 1 at negative potentials and negative values in the positive voltage range. Clearly, these values are not in the valid range of conversion efficiencies (i.e., between 0 and 1).

The reason for this is as follows: As mentioned earlier, η is defined as the useful energy output (i.e., the energy contained in the substrate gradient) divided by the energy input (i.e., the electrochemical potential of Na^+^ and Cl^−^). In Figure 3E and also in earlier figures, we calculated the conversion efficiency as the energy output of the loosely-coupled model divided by the energy output of the fully-coupled model. This is usually a valid approach, because in a fully-coupled model the energy output of the transporter equals its energy input. Yet, in the specific case of the model in Figure 2E the situation is more complicated. The fully-coupled version of the scheme in Figure 2E is that of an electroneutral transporter (i.e., Figure 2C), which cannot harvest the energy contained in the electric field of the membrane. Allowing for slippage, however, converts the electroneutral transporter into an electrogenic transporter, which can utilize the energy contained in the electric field of the membrane. Accordingly, at potentials different from zero, the above definition of η fails, because the energy input of the model with slippage can—dependent upon the voltage chosen—be higher or of opposite sign to the energy input of the fully-coupled transporter. A notable consequence is that the loosely-coupled transporter is predicted to have a higher concentrative power than the fully-coupled transporter at potentials more negative than −70 mV (Figure 3F). We verified that in the model with Cl^−^ slippage, voltage affected the ability of the transporter to harvest the energy contained in the electrochemical potential of Cl^−^ by calculating the “fraction of the Cl^−^ potential used”, i.e., the fraction of the energy converted into substrate translocation: This “fraction of the Cl^−^ potential used” is plotted as a function of voltage in Figure 3G. It is evident from this plot that the extent to which the electrochemical potential of Cl^−^ can be exploited is voltage-dependent. We emphasize that in the model of Figure 2E, the vast majority of the energy is supplied by the electrochemical potential of Na^+^ (e.g., at 0 mV, 6.6 kJ/mol and 0.15 kJ/mol are provided by the chemical potential of Na^+^ and Cl^−^, respectively)

We conclude that the conversion efficiency plays an important role in defining the substrate concentrations at the steady state, i.e., the concentrative power of an ion-coupled transporter. However, at low conversion efficiencies, η is an intricate function of ion and substrate affinities and of the voltage-dependent partial reactions in the transport cycle. In other words, η is defined by the kinetics of a transporter. Hence, insights into the rates governing the transport cycle are a prerequisite for correctly predicting the concentrative power of a partially-coupled transporter. However, it is not possible to access all states of the transport cycle by direct measurements. Hence realistic kinetic models are required to extract the rates for the partial reactions.

### 2.5. How to Build a Credible Kinetic Model of a SLC6 Transporter

Kinetic models have been used to understand the logic of substrate transport through SLC6 transporters [19,21,22,32,33,34]. However, there is no established set of rules, which defines the requirements a credible kinetic model of a SLC6 transporter must meet. Most authors, who employed kinetic models of SLC transporters, restricted themselves to very simplified schemes of the transport cycle. These typically accounted only for a subset of experimental observations [29,30]. In addition, in most cases, limited efforts were made to incorporate the available published findings into the models. A guiding principle of kinetic modeling is parsimony, which favors simplified models.

However, there are important boundary conditions, which the credible kinetic model SLC6 transporters must meet: (i) The model must correctly predict the intra- and extracellular substrate concentrations at steady state; (ii) the model must also account for the known voltage-dependence of partial reactions and (iii) the consistently observed binding cooperativity between co-substrates and substrate. (iv) The output of the model (i.e., the synthetic data) must recapitulate, both experimentally-observed transport rates and electrophysiological recordings. These conditions determine the level of acceptable parsimony.

### 2.6. What is the Minimal Number of States that Allows for Correctly Predicting the Intra- and Extracellular Substrate Concentrations at the Thermodynamic Equilibrium?

**Figure 4:**
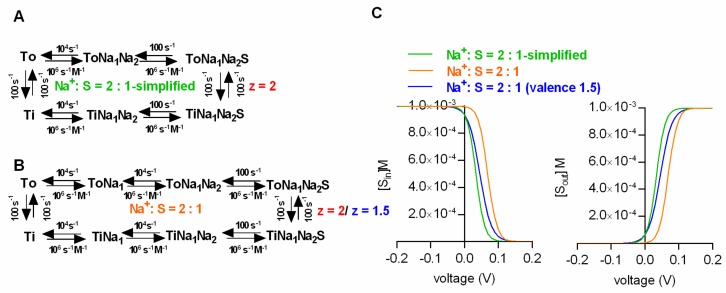
Limits of parsimony. Displayed in (**A**) and (**B**) are the schemes of two kinetic models, which both adhere to a stoichiometry of Na^+^:S = 2:1. In the scheme in **A**, both sodium ions bind in a single transition (i.e., simplified model). In **B**, each sodium ion is allowed to bind individually. (**C**) Intra- and extracellular substrate concentrations at steady state (left and right panel, respectively) for the simplified model in **A** (green trace), the model in **B** (orange trace) and for the model in **B**, but under the assumption that the net-valence does not match the net-charge of the chosen stoichiometry (1.5 vs. 2- blue trace). Only the orange traces match the prediction of the thermodynamic equation in Figure 1A.

Arguably, a credible kinetic model of the transport cycle should also apply once the intra- and extracellular substrate concentrations have reached the thermodynamic equilibrium. Here, we demonstrated that kinetic models can faithfully predict the substrate concentrations at steady state. This, however, is only true, if the binding of each individual co-substrate is explicitly represented in the kinetic scheme. Conversely, if for the sake of parsimony, binding events of co-substrates are lumped together or omitted, the model fails to correctly emulate the inner and outer substrate concentrations at the steady state. This is illustrated in Figure 4, where we compare two kinetic models, which both adhere to a stoichiometry of 2Na^+^/S. In the more parsimonious model (i.e., with fewer states and reaction rates), binding of the 2 Na^+^ ions is assumed to occur in a single transition (Figure 4A). In the more realistic model, the 2 Na^+^ ions are allowed to bind individually (Figure 4B). Importantly, only the latter model accurately predicts the inner and outer substrate concentrations as predefined by the thermodynamic equation (Figure 4C). Hence, if compliance to the rules of thermodynamics is considered an important boundary condition, the model must include the following minimum number of states: (Number ligands + 2) × 2. We note that this minimal number of states can only be realized in a scheme in which co-substrates and substrate are assumed to bind in sequential order.

### 2.7. Incorporation of Voltage Dependence in Models of SLC6 Transporters: Summed Valences must Match the Stoichiometry

Most SLC6 transporters are electrogenic: In each transport cycle one or more net charges are moved through the membrane. As a consequence, a subset of partial reactions in the transport cycle must be voltage-dependent. In kinetic models of SLC6 transporters, the voltage dependence is incorporated by the assignment of valences to those partial reactions that move charge through the membrane. For instance, assigning a valence of 1 to a partial reaction implies that during this reaction, one charge is moved through the entire electric field of the membrane. However, the charge moved in a reaction does not necessarily need to assume an integer value. In fact, where this was experimentally determined, partial reactions often carried a fraction of a net-charge [21,22,34]. In this context, it is also important to point out that reactions, which move ions through the electric field, do not necessarily need to be voltage-dependent. A situation can be envisaged, where a positively-charged molecule is neutralized by complex formation with a negatively-charged amino acid residue on the transporter. The translocation of the neutral complex through the membrane is electrically silent. It is only when the negative charge of the residue returns in a subsequent transition that net charge is moved through the membrane.

In a credible kinetic model of the SLC6 transporter, the assigned valences must sum to the net-charge/cycle, as predicted by the stoichiometry. Compliance to this rule ensures that the model correctly predicts the substrate concentrations at the thermodynamic equilibrium. This is illustrated in Figure 4. Here, we compared two models with a stoichiometry of 2Na^+^/S. One in which the valence summed to the net-charge, as predicted by the stoichiometry (i.e., 2, orange line in Figure 4C) and one where the summed valence was less (i.e., 1.5, blue line in Figure 4C). Only the former correctly emulated the intra- and extracellular substrate concentrations at the thermodynamic equilibrium as predicted by the equation shown in Figure 1A.

### 2.8. Cooperative Binding: A Common Feature of SLC6 Transporters?

For the sake of parsimony, most published kinetic models of SLC6 transporters adhere to a sequential binding order [22,24,34]. The question, however, is whether the assumption of sequential ion and substrate binding to SLC6 transporters is realistic. Structures of LeuT, dDAT and hSERT all show that the Na2 binding site is located distal to the substrate binding site (i.e., when viewed from the extracellular side) [35,36,37]. This layout suggests that the binding order of this Na^+^ and the substrate is sequential (i.e., Na^+^ binding to the outward-facing conformation occurs prior to the substrate, and Na^+^ is the first to dissociate into the cytosol upon conversion of the transporter to the inward-facing conformation). However, when tested by experimental approaches, the binding order of Na^+^ and substrate in SERT was determined to be random and not sequential [15,38].

In this context, it is worth noting that for every SLC6 transporter, where this was explicitly explored, the binding of co-substrates and substrate was found to be cooperative [15,16,39,40]: Co-substrates and substrate bound per se with lower affinity than upon ternary complex formation (i.e., transporter, co-substrates and substrate). An important consequence of this has been frequently overlooked: Cooperativity, which implies that co-substrate and substrate bind in a random order, because a sequential binding scheme cannot account for cooperative co-substrate and substrate binding. We thus believe that the random order binding of co-substrate and substrate is the default situation in SLC6 transporters, rather than the exception. If true, it is a reasonable requirement for a credible kinetic model of the SLC6 transporter to account for this property.

**Figure 5:**
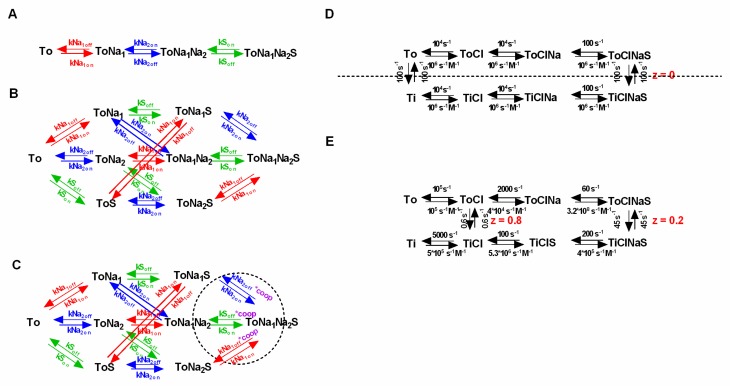
Random order binding of co-substrate and substrate and incorporation of cooperativity. (**A**) and (**B**) Sequential and the corresponding random order binding scheme, respectively. Both schemes adhere to a stoichiometry of Na^+^:S= 2:1. Rates are shown in the same color (i.e., blue, green or red) if they parameterize the binding of the same ligand. (**C**) Cooperative binding scheme. To implement cooperativity a coop factor (coop in magenta) was introduced in the highlighted loops (dashed circle). The coop factor is a number between 0 and 1, which when multiplied with the indicated dissociation rates, renders the model cooperative. (**D**) Example of a symmetric model. Intra—and extracellular—co-substrate and substrate affinities are the same, and the inward- and the outward-facing states are equally stable. The dashed line indicates the axis of symmetry. (**E**) Asymmetric Model of dopamine (DAT) [22]. Shown in the scheme are the published rates and valences.

If random order binding is incorporated into a model of SCL6 transporters, there is a surge in the number of states and reaction rates. The question which then arises is whether such models are overparameterized. In the following we lay out reasons why we think that this is not the case: Figure 5A,B show a sequential and the corresponding random order binding scheme (stoichiometry: Na^+^:S = 2:1). In the random order binding scheme, the reaction rates, which parameterize the binding of an individual co-substrate or the substrate, are expected to be the same, irrespective of whether the binding/unbinding of this specific ligand occurs before or after the binding/unbinding of another co-substrate/substrate.

This is justified, because it is unreasonable to assume that the ligand affinity is contingent on the time point of dissociation/association. In the presented scheme we highlight in color (red, blue and green) the parameters, which according to this argument, have to remain constant, and which are thus constrained. In addition, microscopic reversibility demands that the product of the rates in one direction of a loop is equal to the corresponding product in the opposite direction. It is obvious that there are a multitude of loops in a random order scheme, each nested in other loops. Because, the principle of microscopic reversibility applies to all loops, this greatly restricts the available parameter space. In fact—in a fully random order binding scheme—the two constraints (i.e., time independence and microscopic reversibility) limit the choice of free parameters to exactly the same number required in the sequential binding scheme (see Figure 5A).

### 2.9. How to Incorporate Cooperativity

Above we argued, that it is unreasonable to assume that the ligand affinity is contingent on the time point of dissociation/association in a fully random order scheme. However, this is exactly what appears to be the case in the scenario of cooperative binding. Here, the time point of the binding event seems critical, because the kinetics of ligand binding are expected to be different depending on whether the other ligand has or has not yet associated/dissociated beforehand. However, it is important to understand that in the scenario of cooperative binding, the affinity change is not a function of time, but a consequence of the other ligands being present or absent. An intuitive understanding of this is provided by the available structures. In the structure of LeuT, for instance, the Na^+^ ion at the Na1 site was shown to directly interact with the substrate. Because the Na^+^ ion has bonding interactions with the substrate and vice-versa, the two ligands reciprocally shape their corresponding affinities.

Given the constraints imposed by microscopic reversibility and by the time-independence of ligand binding, the only feasible way to incorporate cooperative binding into a kinetic model is by introducing a “cooperativity factor”. As highlighted in Figure 5C (dashed circle) this cooperativity factor (designated “coop” in magenta) is multiplied with the dissociation constant of each ligand in the indicated loops. This procedure elegantly implements cooperative binding into the kinetic model, while maintaining microscopic reversibility. Importantly, the cooperativity factor is the only required additional parameter. Considering all of the existing constraints, we therefore argue that a random or cooperative binding scheme—despite its complexity compared to the sequential scheme—is not overparameterized. The decision to include cooperative binding into a kinetic model of an SLC6 transporter increases the number of required states to 2 × 2^number of ligands^.

### 2.10. Symmetry in Kinetic Models of SLC6 Transporters

Published kinetic models of SLC6 transporters frequently posit symmetry [16,21]: All binding rates of co-substrates and substrate for the outward- and the inward-facing conformations are assumed to be the same, and the outward- and the inward-facing state are assumed to be equally stable. Metaphorically, a symmetric model of the transport cycle posits that there would not be any noticeable difference—not even at a pre-steady state, if the transporter were extracted from the plasma membrane and reinserted with inverted topology. This seems to be a bold assumption, but, as pointed out earlier, the thermodynamic equation in Figure 1A does not reference the orientation of the transporter. This shows that asymmetry is not a requirement for vectorial substrate uptake. In fact, it is a widespread misconception to posit that substrate must bind to the outward-facing conformation with higher affinity than to the inward-facing conformation to support an inwardly-directed substrate flux. The flaw in this idea is exposed by inspecting the thermodynamic equation (cf. Figure 1A): binding affinities—much like transporter orientation—are immaterial to the concentrative power.

There are arguments for and against symmetry. The symmetric model is more parsimonious than an asymmetric model, because each individual rate in a symmetric model is tied to its mirrored rate (the dashed line in Figure 5D indicates the axis of symmetry). Thus, symmetry avoids overparameterization. Another advantage of a symmetric model is that it naturally fulfills microscopic reversibility. The question, however, is whether the assumption of symmetry is realistic. One could argue, for instance, that affinities are expressed as rational numbers, and the chance that two rational numbers are identical is essentially zero. Moreover, there are also structural arguments against symmetry, such as the fact, for instance, that the inward- and the outward-facing structures of LeuT are by no means identical mirror images [35,41].

It is also possible to maintain microscopic reversibility in an asymmetric model. For instance, Erreger et al. chose a model which posited a higher substrate affinity to the outward- than to the inward-facing conformation of DAT (Figure 5E) [26]. They maintained microscopic reversibility by assuming that Na^+^ bound with higher affinity to the inward- than to the outward-facing conformation. However, it is worth pointing out that Erreger et al. were not compelled by their data to select a model with asymmetric intra- and extracellular affinities for Na^+^ and substrate. In fact, there is a peculiar problem arising from an asymmetric model: If, for instance, a higher substrate affinity is posited for the outward- than for the inward-facing state of an SLC6 transporter, compensatory rate changes ensue in other reactions of the transport cycle to maintain microscopic reversibility. In the above example, the authors chose to change the binding rates of Na^+^ to DAT to the effect that the Na^+^ affinity for the inward-facing state became higher than its affinity to the outward-facing state. However, the question which then arises is: Why would lowering the intracellular substrate affinity increase the intracellular Na^+^ affinity? The fact that this was a necessary adjustment to maintain microscopic reversibility implies a causative link. This to us is a very discomforting aspect of asymmetric models. In conclusion, we think that there are more arguments in favor of adhering to symmetry than there are for asymmetry. We thus advocate for using symmetric models, unless of course, the assumption of symmetry is convincingly contradicted by experimental results.

## 3. Materials and Methods

We built kinetic models of secondary active transporters featuring different stoichiometries. Time-dependent changes in state occupancies were evaluated by numerical integration of the resulting system of differential equations using the Systems Biology Toolbox [42] and MATLAB 2015a (MathWorks). The voltage dependence of individual rates was modeled according to [43] assuming a symmetric barrier as

(1) $$k_{ij}=k_{0,ij}\cdot exp\left(\frac{-z\cdot Q_{ij}\cdot F\cdot V}{2\;R\cdot T}\right)$$

with F = 96,485 with C mol^−1^, R = 8.314 JK^−1^mol^−1^, V as the membrane voltage in volts, T = 293 K, k0,ij are the rates at 0 mV and z·Qij is the net charge transferred during the transition.

Pre-steady state substrate uptake was calculated as:

(2) $$\frac{d}{dt}Nin=\left(TiClNaS\cdot koffSin-TiClNa\cdot konSin\cdot S_{in}\right)\cdot \frac{NC}{NA}$$

This equation applies to the kinetic reaction schemes in Figure 2C,E. NC is the number of transporters, and NA is Avogadro’s Number. N_in_ number of substrate molecules in mol, which enter the cell via the transporter. The expressions koffSin and konSin are the dissociation and association rate constants of the substrate, which parameterize substrate binding to the inward-facing conformation. For the simulation of initial uptake, we assumed that the intracellular substrate concentration (S_in_) is zero.

The substrate concentrations (S_in_ and S_out_) in the intra- and extracellular compartment were calculated as:

(3) $$S_{in}=\frac{N_{in,0}+N_{in}}{V_{in}}$$

(4) $$S_{out}=\frac{N_{out,0}+N_{out}}{V_{out}}$$

(5) $$\frac{d}{dt}Nout=\left(ToClNa\cdot konSout\cdot S_{out}-ToClNaS\cdot koffSout\right)\cdot \frac{NC}{NA}$$

(6) $$\frac{d}{dt}Nin=\left(TiClNaS\cdot koffSin-TiClNa\cdot konSin\cdot S_{in}\right)\cdot \frac{NC}{NA}$$

These equations apply to the kinetic reaction schemes in Figure 2C,E. N_in,0_ and N_out,0_ are the initial number of substrate molecules in mol in the intracellular (with volume V_in_) and extracellular compartment (with volume V_out_), respectively. N_in_ and N_out_ are the number of substrate molecules in mol entering the intra- and extracellular compartments via the transporter, respectively. NC is the number of transporters, NA is Avogadro’s Number (6.022 × 10^23^).

**Figure 6:**
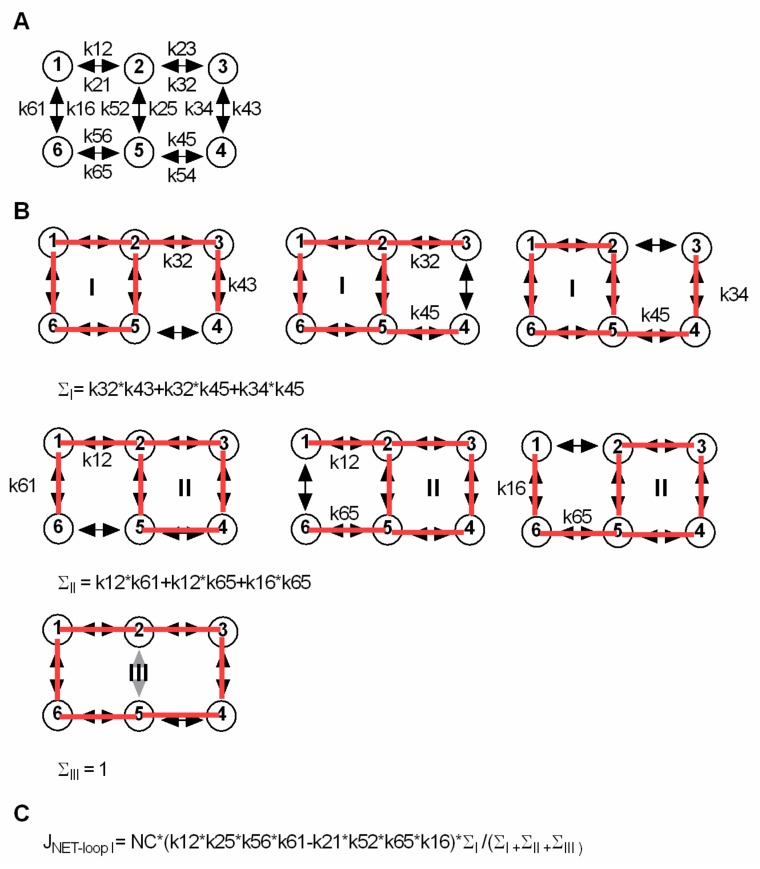
Analytical approach to calculate the intra- and extracellular substrate concentrations at steady state for a kinetic model in which the driving ion and the substrate are loosely coupled. (**A**) Shows a simple scheme, identical to the scheme in Figure 2A. (**B**) Approach to calculate the net flux through a loop within a kinetic model. The scheme has three loops I, II and III. In **B**, we show how to derive the equation for the net flux through loop I (J_NET-loopI_). In the upper row we depict the longest possible paths to enter loop I. Each of these paths is one transition short of closing a second loop. Finding these paths is necessary to obtain ∑I: the rates in each individual path are multiplied, and the rates of different paths are summed. The same approach applies to loop II (middle row). ∑III is 1, because no path exists, which would not immediately close a second loop. (**C**) Equation for the net flux through loop A. NC is the number of transporters, which in the equation is multiplied with the product of the rates in loop I in the forward direction, minus the product of the rates of loop I in the reverse direction. The net flux through loops II and III can be derived in an analogous manner. The intra- and extracellular substrate concentrations at steady state can be found by solving for the substrate concentrations at which the net flux trough loop II equals the net flux through loop III. This is the condition at which there is no net flux of substrate through the transporter (i.e., steady state).

The analytical approach to calculate S_in_ and S_out_ at steady state was described by Hill [5]. The scheme in Figure 6 shows a six-state model identical to the one used in Figure 2A. The six-state model has three loops (A, B and C). In the figure, the approach is explained to calculate the net flux through loop A (JNET-loopA). All possible ways to enter loops A, B and C are drawn always one transition short of closing a second loop. In this way ∑A and ∑B can be determined. ∑C is 1. For calculating S_in_ and S_out_ at steady state, we solved for the intra- and extracellular substrate concentrations at which the sum of the net flux through loop B and loop C is zero, because this is the condition at which there is no net flux of substrate (i.e., steady state).

The energy output of all models with slippage is:

(7) $$E_{output}\left[\frac{J}{mol}\right]=R\cdot T\cdot \ln\frac{S_{in,\;slip}}{S_{out,\;slip}}$$

where R and T are the gas constant and absolute temperature, respectively, and S_in,slip_ and S_out,slip_ are the intra- and extracellular substrate concentrations at steady state predicted by a model, which allows for Na^+^ or Cl^−^ slippage.

The conversion efficiency is defined as:

(8) $$\eta =\frac{E_{output}}{E_{input}}$$

If the electrochemical potential of the ions and the substrate are fully-coupled, E_out_ equals E_in_. We therefore define the conversion efficiency as:

(9) $$\eta =\frac{E_{output}\;of\;the\;model\;with\;slippage}{E_{output}\;of\;the\;fully\;coupled\;model}$$

E_output_ of the fully-coupled model is:

(10) $$E_{output}\left[\frac{J}{mol}\right]=R\cdot T\cdot \ln\frac{S_{in\;}}{S_{out}}$$

where S_in_ and S_out_ are the intra- and extracellular substrate concentrations obtained from the thermodynamic equation in Figure 1A.

In the model in Figure 2E all energy contained in the electrochemical potential of Na^+^ is used to maintain the substrate gradient, while of the electrochemical potential of Cl^−^, only a fraction (**A**) is utilized. **A** can be obtained from the following equation:

(11) $$R\cdot T\cdot \ln\frac{S_{out,\;slip}}{S_{in,\;slip}}=(R\cdot T\cdot \ln\frac{Na_{in}}{Na_{out}}+F\;zNa*VM)+A\cdot (R\cdot T\cdot \ln\frac{Cl_{in}}{Cl_{out}}+F\cdot zCl\cdot VM)$$

We defined the fraction of the Cl^−^ potential used as:

(12) $$A\cdot (R\cdot T\cdot \ln\frac{Cl_{in}}{Cl_{out}}+F\cdot zCl\cdot VM)/(R\cdot T\cdot \ln\frac{Cl_{in}}{Cl_{out}}+F\cdot zCl\cdot VM)$$

where F is the Faraday constant, zNa and zCl are the valences of Na^+^ and Cl^−^ (i.e., + 1 and −1, respectively) and V_M_ is the membrane potential.

## 4. Conclusion

During their transport cycle, SLC6 transporters transit many conformational states; >8 distinct structures, for instance, have been solved for the bacterial betaine transporter BetP [39]. The transition between these conformations can be addressed by molecular dynamics simulations. Kinetic and thermodynamic modeling of the transport cycle provides boundary conditions for these computational approaches to bridge the gap between static structures and dynamic rearrangements. In addition, the exercise of kinetic modeling allows for an understanding of the physiological role of transporters at the macroscopic level. This can be illustrated by pointing out that, based on their thermodynamics, even concentrative transporters function as concentration clamp: They can supply extracellular substrate by running in the reverse mode. In fact, this is the mechanism by which GlyT1 supplies glycine as a co-transmitter [16]. Similarly, it has long been appreciated that monoamine transporters do not only reside in the presynaptic specialization, where they operate in relay with the vesicular monoamine transporters [44]; they have also been visualized on the somatodendritic membrane [45,46]. Somatodendritic transporters can mediate neurotransmitter release [47] and regulate neurotransmission in a manner distinct from presynaptic transporters [48].

## Abbreviations

hSERT	humane serotonin transporter
hDAT	Drosophila dopamine transporter
NET	Noradrenaline transporter
GlyT1	Glycine transporter 1
BetP	Bacterial betaine transporter
SLC	Solute carrier
5-HT	5-hydroxytryptamine
LeuT	Bacterial leucine transporter
